# Comparison of dehulling efficiency and grain nutritional parameters of two cultivated barnyard millet species (*Echinochloa* spp.)

**DOI:** 10.1016/j.heliyon.2023.e21594

**Published:** 2023-10-31

**Authors:** Salej Sood, Tilak Mondal, Ramesh S. Pal, Dinesh C. Joshi, Lakshmi Kant, Arunava Pattanayak

**Affiliations:** a ICAR- Central Potato Research Institute, Shimla, HP, India; b ICAR-Vivekananda Institute of Hill Agriculture, Almora, Uttarakhand, India; c ICAR- Indian Institute of Agricultural Biotechnology, Ranchi, Jharkhand, India

**Keywords:** *Echinochloa frumentacea*, *E*. *esculenta*, Dehulling recovery, Grain physical parameters, Nutritional value, Antioxidant activity

## Abstract

Due to increased awareness regarding the health-promoting profile of millets, inclination towards their consumption has increased considerably. In the Himalayan region of India, cultivars of the two species of barnyard millet, namely Indian (*Echinochloa frumentacea*) and Japanese barnyard millet (*E*. *esculenta*), are grown. To compare the dehulled grain recovery, grain physical parameters, nutritional profile and antioxidant activity, an experiment was carried out at ICAR-VPKAS, Almora, Uttarakhand hills for two years using released and popular cultivars of Indian barnyard millet (VL 207 and VL 172) and Japanese barnyard millet (PRJ-1). The results indicated that the whole grain yield of Japanese barnyard millet cultivar PRJ-1 was significantly higher than Indian Barnyard millet cultivars VL 172 and VL 207; however, the dehulled grain recovery was considerably higher in VL 172 and VL 207 than PRJ-1. Similarly, the physical grain parameters were significantly higher in PRJ-1, but most dehulled grain parameters were at par in cultivars of both species. The nutritional estimation of dehulled grains of both species did not show remarkable differences for most traits. Still, crude fibre, Mn, and Zn were high in PRJ-1, while total digestible nutrients and phosphorous were high in VL 172 and VL 207. Dehulled grains exhibited much more crude protein, ash, minerals, and total digestible nutrients, but the husk accumulated significantly higher crude fibre and total polyphenols.

## 1 Introduction

The genus *Echinochloa* has two cultivated species, *Echinochloa frumentacea* (Indian barnyard millet) and *Echinochloa esculenta* (Japanese barnyard millet), predominantly grown for food and fodder [1]. Both the species of the genus have been cultivated for centuries because of their immense nutritional importance for humans and animals and their ability to withstand low-input agriculture and harsh environmental conditions. *E. frumentacea,* commonly known as Indian barnyard millet, originated in India and showed a parallel line of evolution in India and Africa, while *E. esculenta* (Japanese barnyard millet) is a native of Japan [2]. Indian barnyard millet is best adapted to lowlands, particularly sub-tropical and tropical conditions. In contrast, Japanese barnyard millet is adapted to temperate climates only, and the crop's full potential is realised in hills at an elevation of >1500 m above mean sea level [3]. Both species have independently evolved from wild progenitors and can be distinguished quickly based on their inflorescence morphology [4].

Whole grains of barnyard millet are used as animal feed, while humans consume the kernels [5]. The economic part of barnyard millet is kernel, consumed as rice after dehulling [1]. Dehulling is a tedious process in barnyard millet as lemma and palea are tightly bound to kernels. In temperate climates, Japanese barnyard millet (*E. esculenta*) outperforms Indian barnyard millet (*E. frumentacea*) regarding grain yield; however, dehulling is more challenging with Japanese barnyard millet [6]. Further, both species have remained isolated without mixing gene pools due to hybridisation barriers between them. Thus, it becomes imperative to evaluate the dehulling efficiency of both species in terms of kernel recovery.

The dehulled millet offers essential components of a healthy diet for humans and animals. The grains provide a spectrum of substances such as gluten-free protein, slowly digestible starch, dietary fibre, minerals, other trace elements, and phytochemicals, especially polyphenols [2]. As a result, the consumption of barnyard millet grains and their value-added products has received significantly more global attention. Subsequently, barnyard millet has recently become a superfood in various culinary compositions.

Compared to other major food grains, Indian barnyard millet is generally well-characterized and has superior protein quality, mineral content, and health-promoting bioactive components like flavonoids, tannins, and alkaloids [7]. In contrast, reports about the nutritional characterisation of Japanese barnyard millet are scanty. Therefore, a comparative study regarding the physicochemical parameters of both cultivated species can be valuable to incentivise their cultivation and assess the differences between the cultivars of both species.

Previous studies have reported the influence of dehulling on physicochemical characteristics, nutritional and bioactive components, and antioxidant properties in various minor millets [[8], [9], [10]]. A series of combined physiochemical reactions cause these changes during decortication. Therefore, understanding these changes can contribute to determining the best processing method for the consumption of barnyard millet grains.

Over the years, we have observed thick hulls, i.e., multiple layers of husk tightly bound to kernels, in Japanese barnyard millet compared to Indian barnyard millet. Therefore, the specific hypotheses under consideration were (i) cultivars of Indian and Japanese barnyard millet differ significantly for dehulling efficiency and physicochemical parameters of grains, and (ii) dehulling induced significant changes in grain physicochemical parameters of Indian and Japanese barnyard millet. To our knowledge, there is no comprehensive study regarding the comparative dehulling efficiency of Indian and Japanese barnyard millet species. Furthermore, information regarding the physical parameters, chemical characteristics, mineral content and antioxidant properties of Indian and Japanese barnyard millet cultivars is scanty. In this context, this study could be of great interest for enhancing nutritional quality, food processing and value addition of this important, underutilised crop.

## 2 Materials and methods

### 2.1 Plant material

The barnyard millet crop was grown at the experimental farm of ICAR-Vivekananda Institute of Hill Agriculture, Almora (79.39° E longitude and 25.35° N latitude, mean rainfall-1000 mm and 1250 m amsl) during the rainy season of 2014 and 2015 following standard agronomic practices. Two Indian barnyard millet cultivars (VL 172 and VL 207) with different genetic backgrounds and one Japanese barnyard millet cultivar (PRJ-1) were included in the study. The Japanese barnyard millet cultivar (PRJ-1) is specifically adapted to the hill agroecosystem and derived through pure-line selection from a Japanese barnyard millet germplasm line IEc 542. All three cultivars are widely adapted and popular among the farmers. The whole grain yield was recorded on a plot basis (3.375 m^2^) and converted into kg per hectare. Five rows of each cultivar were planted in entry row order in three replications during both years. The row length was 3 m with row-to-row spacing of 22.5 cm. Plots were initially over-planted and thinned later during the first weeding to maintain plant-to-plant spacing of 10 cm within the rows.

### 2.2 Dehulled grain recovery and grain physical parameters

Dehulling was performed utilising the VL Millet Thresher. The grains were air-dried to ∼10 % moisture content before dehulling. VL Millet Thresher was developed at ICAR-Vivekananda Institute of Hill Agriculture, Almora, Uttarakhand, India, to thresh and dehull barnyard millet grains easily. The machine has a dehulling capacity of 2.5–4.0 kg barnyard millet grains per h [11]. An equal amount (5 kg) of freshly harvested grains of all three varieties was dehulled using VL Millet thresher for two consecutive years, 2014 and 2015. The grains were passed four times through VL Millet Thresher for dehulling. The following formula calculated the per cent dehulled grain recovery:

Dehulledgrainsrecovery(%)=(Dehulledgrainsweight/Wholegrainsweight)×100

The morphological parameters (length, breadth and thickness) of whole and dehulled grains were measured using a digital vernier calliper with an accuracy of 0.01 mm. The moisture content in grains and kernels was recorded using a moisture meter (FARMEX, USA). The grain physical parameters like bulk density, true density, bulk porosity, arithmetic and geometric mean diameter, sphericity, surface area and aspect ratio were computed following the method described by Ukey et al. [12] and Unal et al. [13].

### 2.3 Proximate analysis and nutrient profiling

For proximate analysis, dehulled grains and husk were air-dried until the dry matter weight became constant. The moisture content was determined by drying the sample at 55 °C to a constant weight. The difference between the fresh and dry weight was used to calculate the moisture content of the dehulled grains and husk.

The dry matter percentage was calculated using the following formula

Drymatter(DM%)=(Dryweightofthesample/Freshweightofthesample)×100

Dry Matter (DM), Crude Protein (CP), Crude Fibre (CF), Ether Extract or Crude Fat (EE), Crude Ash (CA), Acid Detergent Fibre (ADF) and Neutral Detergent Fibre (NDF) of the samples were determined according to AOAC [14]. The Micro Kjeldahl method was followed for nitrogen determination [15]. Briefly, oven-dried samples were digested with H_2_SO_4_ in a catalyst mixture containing K_2_SO_4_ and CuSO_4_. A known aliquot of the diluted sample was distilled in 10 ml of 2 % boric acid solution and titrated against standard 0.1 N H_2_SO_4_. Crude protein was calculated by multiplying the nitrogen content with a conversion factor 6.25. The EE content in a sample was determined by extracting with diethyl ether at 60 °C in Auto-Soxhlet's apparatus (Pelican socsplus- SCS 02AS). For CF, samples were reflexed first with 1.25 % H_2_SO_4_ and subsequently with 1.25 % NaOH for 30 min each to dissolve acid and alkali-soluble components. The residue containing CF was dried to a constant weight, and the dried residue was ignited in a muffle furnace; loss of weight on ignition was calculated to express it as CF. For CA, samples were ignited in a muffle furnace at 550 °C to burn all the organic matter, and the leftover was weighed as ash. Acid detergent fibre (ADF) was determined by refluxing 1.0g of sample with 100 ml ADF extraction reagent for 1 h. The solution was filtered, washed, dried overnight at 100 °C and weighed. NDF was determined by refluxing 1g sample with 100 ml NDF extraction reagent for 1 h. The solution was filtered, residue washed with hot distilled water and then with acetone, dried overnight at 100 °C and weighed. Besides these parameters, additional parameters such as digestible dry matter (DDM), digestible crude protein (DCP), total organic matter (TOM), total carbohydrates (TC), total digestible nutrient (TDN) and nitrogen-free extract (NFE) were calculated on dry matter basis as described by Mondal et al. [16].

1. DDM = 88.9 - (ADF × 0.779)
2. DCP = 0.916 × (CP-3.09)
3. TOM = NFE + CF + EE + CP
4. TC= NFE + CF
5. TDN = 96.35 – (ADF × 1.15)
6. NFE (%) = 100 - (CP (%) + CF (%) + EE (%) + ash (%))

The mineral elements in the kernel and husk, i.e., phosphorus (P), calcium (Ca), iron (Fe), manganese (Mn), copper (Cu) and zinc (Zn), were determined by digesting the samples with di-acid, followed by filtering the residue and making the final volume to 50 mL by adding distilled water. The observations were recorded using AAS (Vario-6, Analytic Jena) for all the mineral elements except Ca, which was recorded using a flame photometer (ELICO- CL378).

### 2.4 Antioxidant metabolite and activities

Each sample (1.0g) was extracted by adding 20 ml 80 % methanol by continuous stirring at 25 °C, 150 rpm/min for 12 h, and filtered through Whatman filter paper No. 1. The extract solution stored at 4 °C in amber bottles served as the working solution (20 mg/ml) to determine total phenolic content and antioxidant activity.

### 2.5 Determination of total polyphenols

Total polyphenol content (TPC) was estimated by a colorimetric assay based on procedures described by Singleton and Rossi with some modifications [17]. Initially, 1.0 ml of methanolic extract (20 mg/ml) was mixed with Folin and Ciocalteu's phenol reagent (1.0 ml). After 3 min, 1.0 ml of a saturated sodium carbonate solution was added to the mixture, and the volume was adjusted to 10 ml with distilled water. The reaction was kept in the dark for 90 min, after which the absorbance was read at 725 nm (Thermo scientific chemitospectra scan UV 2600 spectrophotometer). Gallic acid was used to calculate the standard curve (1–80 μg/ml). The results were mean values ± standard deviations and expressed as mg of gallic acid equivalent (GAE)/g of extract.

### 2.6 Determination of radical scavenging activity on DPPH and ABTS

Radical scavenging activity (RSA) on DPPH was determined by measuring the decrease in absorbance of methanolic DPPH solution at 515 nm in the presence of the extract [18] with some modifications. The samples' methanolic extract (150 μL) was allowed to react with 2850 μL of DPPH working solution for 24h in the dark, and absorbance was recorded at 515 nm. RSA on ABTS was determined by measuring the decrease in absorbance of methanolic ABTS solution at 745 nm in the presence of the extract [19]. Sample extracts (200 μL) were allowed to react with 2000 μL of the freshly prepared ABTS solution for 30 min in dark conditions, and absorbance was taken at 745 nm. Trolox was employed as a reference. DPPH and ABTS radical scavenging activity were expressed in μM Trolox equivalents (TE)/g dry weight.

### 2.7 Determination of total antioxidant activity

The total antioxidant activity (TA) was estimated using the phospho-molybdenum method [20] based on the reduction of Mo (VI) to Mo (V) by the sample analyte and subsequent formation of specific green phosphate/Mo (V) compounds. A 0.3 ml aliquot of extract solution (5–25 mg/ml) combined with 2.7 ml of the reagent solution (0.6 M sulfuric acid, 28 mM sodium phosphate and 4 mM ammonium molybdate) was capped and incubated in a boiling water bath at 95 °C for 90 min. Samples were allowed to cool at room temperature, and absorbance was measured at 695 nm. For the blank, 0.3 ml methanol/double distilled water was mixed with 2.7 ml of the reagent. A standard curve of trolox (10–100 μM) was prepared, and total antioxidant activity was expressed as μM Trolox equivalent/g dry weight (μM TE/g DW).

### 2.8 Statistical analysis

The mean of observations recorded in triplicate on dehulled grain recovery, proximate analysis, and various grain physical and nutritional parameters were pairwise compared using Tukey's HSD test for significant differences among varieties [21]. The mean values of all the observations and standard errors are presented in the tables.

## 3 Results

### 3.1 Dehulled grains recovery and grain physical parameters

**Fig. 1:**
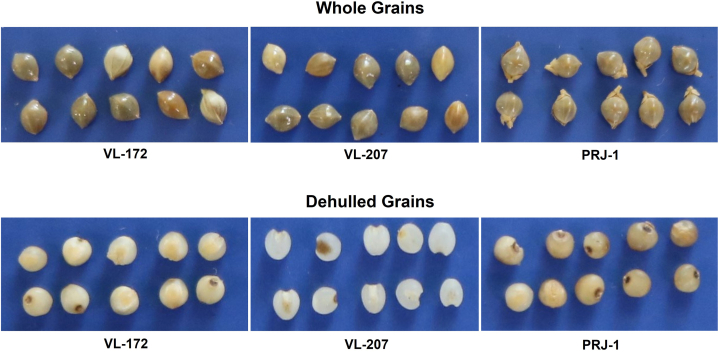
Whole and dehulled grains of barnyard millet cultivars used in the study.

The whole grain yield of the Japanese barnyard millet cultivar PRJ-1 was significantly higher than both the Indian barnyard millet cultivars, VL 172 and VL 207 (Table 1; Fig. 1). The dehulled grains recovery varied from 40.18 ± 0.97 to 55.25 ± 3.19% in both the species. The dehulled grains recovery was significantly lower in *E. esculenta* cv. PRJ-1 (40.18–43.42 %) in comparison to *E. frumentacea* cv. VL 207 (48.83–55.23 %) and *E. frumentacea* cv. VL 172 (46.75–47.57 %) (Table 1). In both evaluation years, the Indian barnyard millet cultivars VL 207 and VL 172 consistently showed significantly higher grain recovery than the Japanese cultivar PRJ-1. Physical measurements of whole grains revealed that cultivar PRJ-1 had significantly larger dimensions than VL 207 and VL 172 except for grain sphericity (Table 1). In contrast, whole-grain sphericity of PRJ 1 was significantly lower than that of VL 207 and VL 172. Similar results were found for the dehulled grains, where the Japanese barnyard millet cultivar PRJ-1 had considerably higher grain weight, grain volume, bulk density, and true density than the Indian barnyard millet cultivars VL 207 and VL 172. All other grain physical parameters were statistically at par for dehulled grains in Indian and Japanese barnyard millet cultivars (Table 1).

**Table 1:** Dehulled grains recovery and grain physical parameters of Japanese barnyard millet and Indian barnyard millet varieties.

Parameters	2014			2015		
	PRJ-1	VL 207	VL 172	PRJ-1	VL 207	VL 172
WGY (kg/ha)	2888 ± 104^a^	2484 ± 98^c^	2680 ± 91^b^	2912 ± 103^a^	2634 ± 98^b^	2564 ± 92^b^
DGR (%)	43.42 ± 1.21^c^	55.25 ± 3.19^a^	46.75 ± 1.34^b^	40.18 ± 0.97^b^	48.83 ± 0.67^a^	47.57 ± 2.01^a^
WGL (mm)	2.71 ± 0.18^a^	2.36 ± 0.09^b^	2.31 ± 0.09^b^	2.62 ± 0.09^a^	2.13 ± 0.06^b^	2.23 ± 0.07^b^
DGL (mm)	1.62 ± 0.08^a^	1.63 ± 0.02^a^	1.54 ± 0.06^a^	1.52 ± 0.13^a^	1.48 ± 0.07^a^	1.49 ± 0.04^a^
WGB (mm)	1.70 ± 0.15^a^	1.52 ± 0.13^b^	1.60 ± 0.06^b^	1.68 ± 0.04^a^	1.60 ± 0.15^b^	1.64 ± 0.09^b^
DGB (mm)	1.56 ± 0.07^a^	1.46 ± 0.02^a^	1.51 ± 0.05^a^	1.51 ± 0.03^a^	1.53 ± 0.03^a^	1.57 ± 0.06^a^
WGT (mm)	1.49 ± 0.05^a^	1.45 ± 0.05^a^	1.42 ± 0.05^a^	1.41 ± 0.04^a^	1.43 ± 0.07^a^	1.44 ± 0.08^a^
DGT (mm)	1.05 ± 0.03^a^	1.06 ± 0.02^a^	0.99 ± 0.03^a^	1.00 ± 0.05^a^	0.98 ± 0.02^a^	0.97 ± 0.02^a^
WGM (%)	10.03 ± 0.03^b^	10.33 ± 0.07^a^	9.78 ± 0.05^c^	9.04 ± 0.08^b^	9.78 ± 0.10^a^	9.62 ± 0.20^a^
DGM (%)	10.03 ± 0.04^b^	15.47 ± 0.03^a^	15.03 ± 0.9^a^	15.37 ± 0.57^a^	14.83 ± 0.02^a^	14.94 ± 0.03^a^
WGW (g)	4.01 ± 0.07^a^	3.01 ± 0.05^b^	3.16 ± 0.04^b^	4.21 ± 0.05^a^	3.20 ± 0.02^b^	3.21 ± 0.03^b^
DGW (g)	3.03 ± 0.08^a^	2.17 ± 0.07^b^	2.11 ± 0.04^b^	2.98 ± 0.07^a^	2.10 ± 0.04^b^	2.15 ± 0.06^b^
WGBD (kg/m^3^)	765 ± 23.10^a^	578 ± 24.30^b^	585 ± 22.80^b^	735 ± 19.10^a^	588 ± 16.30^b^	615 ± 17.80^b^
DGBD (kg/m^3^)	917 ± 18.2^a^	770 ± 11.00^b^	791 ± 27.15^b^	943 ± 11.7^a^	798 ± 18.50^b^	819 ± 21.10^b^
WGTD (kg/m^3^)	1479 ± 35.10^a^	1269 ± 28.23^b^	1290 ± 23.50^b^	1437 ± 27.4^a^	1304 ± 18.9^b^	1270 ± 33.7^b^
DGTD (kg/m^3^)	1580 ± 26.9^a^	1403 ± 28.1^b^	1387 ± 24.6^b^	1630 ± 38.2^a^	1470 ± 33.3^b^	1410 ± 37.4^b^
WGBP (%)	49.25 ± 2.54^a^	55.00 ± 4.90^a^	52.54 ± 3.47^a^	48.87 ± 2.87^a^	54.90 ± 3.64^a^	51.84 ± 2.07^a^
DGBP (%)	40.70 ± 2.08^a^	44.27 ± 3.10^a^	42.98 ± 2.46^a^	42.14 ± 1.95^a^	45.68 ± 2.20^a^	41.88 ± 1.87^a^
WGAMD (mm)	1.97 ± 0.05^a^	1.78 ± 0.07^b^	1.78 ± 0.04^b^	1.9 ± 0.03^a^	1.72 ± 0.05^b^	1.77 ± 0.07^b^
DGAMD (mm)	1.41 ± 0.06^a^	1.38 ± 0.08^a^	1.35 ± 0.06^a^	1.34 ± 0.04^a^	1.33 ± 0.07^a^	1.34 ± 0.05^a^
WGGMD (mm)	1.9 ± 0.08^a^	1.73 ± 0.07^b^	1.74 ± 0.05^b^	1.84 ± 0.06^a^	1.70 ± 0.07^b^	1.74 ± 0.08^b^
DGGMD (mm)	1.38 ± 0.04^a^	1.36 ± 0.05^a^	1.32 ± 0.03^a^	1.32 ± 0.04^a^	1.30 ± 0.03^a^	1.31 ± 0.02^a^
WGS (%)	70.13 ± 1.45^b^	73.42 ± 1.60^a^	75.23 ± 2.54^a^	70.14 ± 2.67^b^	79.60 ± 1.96^a^	78.02 ± 1.79^a^
DGS (%)	85.46 ± 2.17^a^	83.51 ± 2.34^a^	85.74 ± 2.04^a^	86.78 ± 2.23^a^	88.13 ± 2.18^a^	88.19 ± 2.35^a^
WGSA (cm^2^)	11.36 ± 0.29^a^	9.44 ± 0.34^b^	9.5 ± 0.21^b^	10.62 ± 0.39^a^	9.04 ± 0.18^b^	9.52 ± 0.17^b^
DGSA (cm^2^)	6.03 ± 0.14^a^	5.83 ± 0.19^a^	5.48 ± 0.13^b^	5.47 ± 0.16^a^	5.35 ± 0.25^a^	5.43 ± 0.24^a^
WGAR (%)	0.63 ± 0.02^a^	0.64 ± 0.04^a^	0.69 ± 0.03^a^	0.64 ± 0.03^b^	0.75 ± 0.03^a^	0.74 ± 0.03^a^
DGAR (%)	0.96 ± 0.03^a^	0.90 ± 0.05^a^	0.98 ± 0.04^a^	0.99 ± 0.04^a^	1.03 ± 0.05^a^	1.05 ± 0.02^a^
WGSV (mm^3^)	3.6 ± 0.07^a^	2.73 ± 0.05^b^	2.75 ± 0.07^b^	3.25 ± 0.08^a^	2.55 ± 0.05^b^	2.76 ± 0.06^b^
DGSV (mm^3^)	1.39 ± 0.05^a^	1.32 ± 0.04^a^	1.21 ± 0.03^b^	1.20 ± 0.04^a^	1.16 ± 0.03^a^	1.19 ± 0.04^a^

WGY-Whole grain yield; DGR- Dehulled grains recovery; WGL- Whole grain length; DGL- Dehulled grains length; WGB- Whole grain Breadth; DGB- Dehulled grains Breadth; WGT- Whole grain thickness; DGT- Dehulled grains thickness; WGM- Whole grain moisture; DGM- Dehulled grains moisture; WGW-1000-whole grain weight; DGW-1000-dehulled grain weight; WGBD- Whole grain bulk density; DGBD- Dehulled grain bulk density; WGTD- Whole grain true density; DGTD- Dehulled grain true density; WGBP- Whole grain bulk porosity; DGBP- Dehulled grain bulk porosity; WGAMD- Whole grain arithmetic mean diameter; DGAMD- Dehulled grain arithmetic mean diameter; WGGMD-Whole grain geometric mean diameter; DGGMD- Dehulled grain geometric mean diameter; WGS- Whole grain sphericity; DGS- Dehulled grain sphericity; WGSA- Whole grain surface area; DGSA- Dehulled grain surface area; WGAR- Whole grain aspect ratio; DGAR- Dehulled grain aspect ratio; WGSV- Whole grain seed volume; DGSV- Dehulled grain seed volume.

All values with the same superscript, either a or b, are statistically similar; however, a and b are significantly different.

### 3.2 Proximate composition of dehulled grains and husk of Indian and Japanese barnyard millet

The dry matter was high in the husk in comparison to dehulled grains. Compared to the Japanese barnyard millet cultivar PRJ-1, the dry matter content of the dehulled grains and husk of the Indian barnyard millet cultivars VL 172 and VL 207 was higher. However, the differences were not statistically significant. (Table 2). The results were similar for digestible dry matter. The crude protein content was significantly higher in dehulled grains than in husk, but the differences among cultivars in each category, i.e., dehulled grains and husk, were non-significant. Digestible crude protein also observed a similar trend. The ether extract or crude fat varied from 2.00 ± 0.50 (PRJ-1) to 3.33 ± 0.29 (VL 172) and 0.40 ± 0.06 (PRJ-1) to 0.75 ± 0.07 (VL 207) in dehulled grains and husk, respectively. Crude fibre content was significantly higher in PRJ-1 (dehulled grains-9.27 ± 0.31, husk-15.23 ± 0.55) in comparison to both the varieties of Indian barnyard millet, VL 207 (dehulled grains-7.40 ± 0.2, husk-13.10 ± 0.3) and VL 172 (dehulled grains-6.47 ± 0.21, husk-11.23 ± 0.35). The crude fibre was higher in husk than in dehulled grains, while the opposite was true for crude ash (Table 2). Husk had a higher nitrogen-free extract (NFE) concentration than dehulled grains, but there was no statistically significant difference between the cultivars. Similarly, variation in total carbohydrate (TC) content between the Japanese cultivar PRJ-1 and the Indian cultivar VL 207 of barnyard millet was negligible for both the husk and the dehulled grains. Neutral detergent fibre (NDF) was significantly higher in husk than in dehulled grains, and the Japanese barnyard millet cultivar PRJ-1 recorded higher NDF (dehulled grains-27.73 ± 1.72, husk-61.70 ± 1.15) than VL 207 (dehulled grains-24.23 ± 0.45, husk-58.27 ± 1.14) and VL 172 (dehulled grains-23.37 ± 0.49, husk-55.80 ± 0.6) in both the dehulled grains and husk samples (Table 2). A similar trend was observed for acid detergent fibre (ADF), where again PRJ-1 had high values in comparison to VL 207 and VL 172 in both dehulled grains and husk (Table 2). The total digestible nutrients (TDN) were highest in Indian barnyard millet cultivar VL 172 (dehulled grains-83.43 ± 0.40, husk-61.62 ± 1.31), followed by VL 207 (dehulled grains-82.44 ± 0.34, husk-57.29 ± 0.46) and least in PRJ-1 (dehulled grains-79.98 ± 0.52, husk-51.19 ± 0.35). The differences between Indian and Japanese barnyard millet cultivars were significant for TDN.

**Table 2:** Proximate composition of dehulled grains and husk of Indian vs. Japanese barnyard millet cultivars.

	Dehulled grains			Husk		
	PRJ1	VL207	VL172	PRJ1	VL207	VL172
DM	88.69 ± 0.44^a^	90.63 ± 0.14^a^	90.95 ± 0.54^a^	92.64 ± 1.33^a^	94.25 ± 0.78^a^	95.56 ± 0.68^a^
DDM	87.34 ± 0.39^a^	87.21 ± 0.22^a^	86.30 ± 0.22^a^	88.59 ± 0.05^a^	88.32 ± 0.05^a^	88.45 ± 0.02^a^
TOM	94.47 ± 0.35^a^	95.50 ± 0.17^a^	93.53 ± 0.21^a^	99.33 ± 0.12^a^	99.37 ± 0.15^a^	98.97 ± 0.15^a^
CA	5.53 ± 0.35^a^	4.50 ± 0.17^a^	6.47 ± 0.21^a^	0.67 ± 0.12^a^	0.63 ± 0.15^a^	1.03 ± 0.15^a^
CP	9.19 ± 0.06^a^	9.31 ± 0.06^a^	9.47 ± 0.03^a^	2.42 ± 0.04^a^	2.53 ± 0.02^a^	2.65 ± 0.07^a^
DCP	4.41 ± 0.06^a^	4.52 ± 0.06^a^	4.67 ± 0.03^a^	1.21 ± 0.03^a^	1.10 ± 0.02^a^	1.09 ± 0.07^a^
EE	2.00 ± 0.50^a^	2.17 ± 0.29^a^	3.33 ± 0.29^b^	0.40 ± 0.06^a^	0.75 ± 0.07^b^	0.58 ± 0.03^a^
CF	9.27 ± 0.31^a^	7.40 ± 0.2^b^	6.47 ± 0.21^b^	15.23 ± 0.55^a^	13.10 ± 0.3^b^	11.23 ± 0.35^b^
NFE	75.01 ± 0.7^a^	77.62 ± 0.28^a^	75.27 ± 0.63^a^	83.28 ± 0.75^a^	84.98 ± 0.27^a^	86.50 ± 0.54^a^
TC	84.28 ± 0.41^a^	85.02 ± 0.23^a^	81.73 ± 0.44^b^	98.52 ± 0.2^a^	98.08 ± 0.08^a^	97.73 ± 0.18^a^
ADF	14.23 ± 0.45^a^	12.10 ± 0.3^b^	11.23 ± 0.35^b^	39.27 ± 0.31^a^	33.97 ± 0.4^b^	30.20 ± 1.14^b^
NDF	27.73 ± 1.72^a^	24.23 ± 0.45^a^	23.37 ± 0.49^a^	61.70 ± 1.15^a^	58.27 ± 1.14^a^	55.80 ± 0.6^a^
TDN	79.98 ± 0.52^a^	82.44 ± 0.34^a^	83.43 ± 0.40^a^	51.19 ± 0.35^a^	57.29 ± 0.46^b^	61.62 ± 1.31c

DM-Dry Matter, DDM- Digestible Dry Matter, TOM- Total Organic Matter, CA- Crude Ash, *C*P-Crude Protein, DCP- Digestible Crude Protein, EE- Ether Extract or Crude Fat, CF-Crude Fiber, NFE- Nitrogen Free Extract, TC- Total Carbohydrates, ADF-Acid Detergent Fiber, NDF-Neutral Detergent Fiber, TDN-Total Digestible Nutrient.

All values with the same superscript, either a or b, are statistically similar; however, a and b are significantly different.

### 3.3 Macro and micronutrients in dehulled grains and husk of Indian vs. Japanese barnyard millet cultivars

The variation of grain macro and micronutrient data in three barnyard millet cultivars is presented in Table 3. In dehulled grains, the nitrogen, phosphorous, calcium, iron, manganese, zinc and copper content (mg/100g) varied from 1310.0 to 1354.8, 38.30–81.0, 31.62–42.80, 13.0–17.4, 8.87–11.79, 1.39–1.77, 0.24–0.38, respectively. The phosphorous content was significantly higher in Indian barnyard millet cultivars, with a mean value of 51.70 and 81.00 mg/10g in VL 207 and VL 172, respectively. On the other hand, manganese content was significantly higher in the Japanese barnyard millet cultivar (PRJ-1), with a mean value of 11.79 mg/100g. In addition, the nitrogen content in husk was significantly higher in Indian barnyard millet cultivars VL 172 (104.40 mg/10g) and VL 207 (85.50 mg/100g) in comparison to the Japanese barnyard millet cultivar PRJ-1 (66.70 mg/100g). In general, the concentrations of micronutrients, iron, zinc and copper were significantly higher in dehulled grains than in the husk of all three cultivars. However, no significant difference was observed for micronutrient concentration (Fe, Zn and Cu) between the cultivars of Indian and Japanese barnyard millet either in the dehulled grains or in the husk.

**Table 3:** Micro-nutrients in dehulled grains and husk of Indian vs. Japanese barnyard millet cultivars (mg/100g).

Parameter	Dehulled grains			Husk		
	PRJ1	VL207	VL172	PRJ1	VL207	VL172
N	1310.00 ± 10.0^a^	1330.00 ± 10.0^a^	1354.80 ± 5.0^a^	66.70 ± 5.6^a^	85.50 ± 4.0^b^	104.40 ± 11.6^c^
P	38.30 ± 2.5^a^	51.70 ± 3.5^b^	81.00 ± 3.0^c^	3.00 ± 0.2^a^	5.60 ± 1.2^b^	7.90 ± 0.2^c^
Ca	42.54 ± 17.0^a^	42.80 ± 14.6^a^	31.62 ± 11.1^a^	0.41 ± 0.7^a^	0.65 ± 0.5^b^	1.08 ± 0.5^c^
Fe	13.00 ± 0.5^a^	13.40 ± 0.7^a^	17.40 ± 1.0^a^	0.60 ± 0.1^a^	0.60 ± 0.1^a^	1.00 ± 0.1^b^
Mn	11.79 ± 0.41^a^	8.87 ± 0.7^b^	9.80 ± 0.5^b^	0.30 ± 0.02^a^	0.21 ± 0.01^c^	0.25 ± 0.04^b^
Zn	1.77 ± 0.04^a^	1.39 ± 0.1^a^	1.58 ± 0.07^a^	0.09 ± 0.00^a^	0.08 ± 0.03^a^	0.07 ± 0.004^a^
Cu	0.24 ± 0.01^a^	0.38 ± 0.01^a^	0.30 ± 0.01^a^	0.01 ± 0.00^a^	0.01 ± 0.01^a^	0.06 ± 0.01^b^

*N*-Nitrogen; *P*- Phosphorous; Ca- Calcium; Fe-Iron; Mn- Manganese; Cu- Copper.

All values with the same superscript, either a or b, are statistically similar; however, a and b are significantly different.

### 3.4 Antioxidant metabolites and activities

**Fig. 2:**
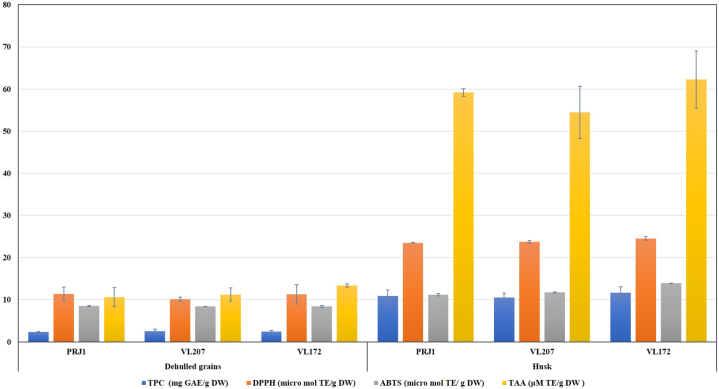
Antioxidants in dehulled grains and husk of Indian vs. Japanese barnyard millet cultivars TPC- Total Polyphenol Content; DPPH- 2,2-Diphenyl-1-picrylhydrazyl; ABTS- 2,2′-azino-bis(3-ethylbenzothiazoline-6-sulfonic acid); TAA- Total Antioxidant Activity.

The amount of total phenolics content (TPC) in the husk was significantly higher than dehulled grains and varied from 2.32 to 2.49 GAE/g DW and 10.52–11.64 GAE/g DW for dehulled samples and husk, respectively. However, the differences between cultivars were non-significant for both dehulled grains and husk independently (Fig. 2). The radical scavenging activity (RSA) of barnyard millet samples was tested against the DPPH and ABTS free radicals, respectively. Like TPC, RSA on DPPH and ABTS was also significantly higher in husk than the dehulled grains of all three varieties. There were no significant differences among the cultivars of Indian and Japanese barnyard millet for the free radical scavenging activity. Similarly, total antioxidant activity (TAA) in the husk of barnyard millet was found to be significantly higher than dehulled grains and varied from 10.62 to 13.39 and 54.43–62.26 μM TE/g DW for dehulled grains and husk, respectively.

## 4 Discussion

Among the various biological factors, moisture plays a vital role in the processing of grains. An optimum moisture level of 10 % has been suggested to effectively process barnyard millet grains [22]; therefore, we dried the whole grains to 10 % moisture content to compare the dehulling efficiency of cultivars of both species. Dehulled grains recovery was higher in both the Indian barnyard millet cultivars (VL 207 and VL 172) compared to the Japanese barnyard millet cultivar (PRJ-1) during both the years of investigation. Interestingly, husk weight was higher in PRJ-1 than VL 207 and VL 172 in both years, but the differences were non-significant. The reasons for more layers of husk in Japanese barnyard millet cultivars' grain are unknown and need further investigation. The plausible reason for thick seed coat in Japanese barnyard millet could be its adaptation in temperate areas, as impermeable thick seed coats have been reported as one of the critical factors for determining freezing avoidance in plant species adapted to temperate areas [23].

The dehulled grains of barnyard millet are the most important economic part of the plant and consumed as a substitute for rice. Therefore, dehulled grain recovery, which ultimately determines the net yield, is an important trait under consideration in breeding programmes. The cultivar PRJ-1 of *E. esculenta* recorded high whole-grain yield (2888 and 2912 kg/ha) in both the years of evaluation in comparison to *E. frumentacea* cultivars VL 207 (2484 and 2634 kg/ha) and VL 172 (2680 and 2564 kg/ha). However, the mean of two years revealed that the Indian barnyard millet cultivars VL 207 and VL 172 had 10.24 % and 5.36 % higher dehulled grains than the Japanese barnyard millet cultivar PRJ-1. This could be one of the reasons that Indian barnyard millet cultivars occupy major area in the hills and are preferred by farmers.

There was no significant difference between the grain physical parameters of dehulled grains of the three barnyard millet cultivars. However, whole grains of the Japanese barnyard millet cultivar PRJ-1 were significantly longer, wider and thicker than the Indian barnyard millet cultivars VL 207 and VL 172. These differences in whole grains of Japanese and Indian barnyard millet cultivars may be attributed to individual properties of species and their adaptability to different growing conditions. Differences in grain physical characteristics of two different sets of buckwheat cultivars, commercial and indigenous genotypes of Turkey, were also observed by Unal et al. [13]. In general, the physical parameters of whole grains were significantly higher than dehulled grains of Indian and Japanese barnyard millet cultivars. This may be attributed to the fact that the dehulling operation removed the outer layer, resulting in grain dimensions reduction. Our findings are consistent with those reported by Dayakar Rao et al. [9], who claimed that dehulling is one of several processing methods that causes significant changes in the physical characteristics of tiny millet grains. The comparative account of grain physical parameters generated for two cultivated barnyard millet species in this study provided a valuable dataset for engineers to design suitable post-harvest technology for drudgery reduction and rapid commercialization of barnyard millet cultivars.

Humans eat dehulled grains, but the husk and whole grains are fed to animals; therefore, we studied the proximate composition of the husk and dehulled grains of Indian and Japanese barnyard millet cultivars. Protein, carbohydrates and fibre constitute the major components of millet seeds. However, the distribution of these components varies within the seed. In the present investigation, the husk fraction of all three barnyard millet cultivars was reported to have a high concentration of total carbohydrate, crude fibre, acid detergent fibre and neutral detergent fibre (Table 2). The concentration of carbohydrates was reduced by 14.24, 13.01 and 16% in PRJ-1, VL 207 and VL 172, respectively (Table 2) upon dehulling. The results agree with previous findings [7] that the outer layer of millet grains (husk) is a rich source of carbohydrates. On the other hand, dehulled grains exhibited high crude protein and total digestible nutrient content in all three cultivars compared to husk (Table 2). High protein concentration in dehulled grains agrees with results reported for buckwheat [24]. However, a reduction in the concentration of protein and fat has been reported in decorticated compared to whole finger millet grains [25]. The primary reason for the high protein content in barnyard millet and buckwheat dehulled grains is the removal of thick seed coat mainly composed of carbohydrates, whereas finger millet grains lack such seed coating. The high concentration of protein reported in dehulled grains of barnyard millet cultivars is advantageous due to the high biological value of the barnyard millet protein. Like crude protein, a higher concentration of crude ash was reported in dehulled grains of barnyard millet in comparison to the husk. Our results correspond to data published by Dizadek et al. [24], which reported a higher ash concentration in dehulled buckwheat grains than the hull. Unlike crude protein and ash in the husk, the dehulled grains had reduced crude fibre content in all three cultivars of barnyard millet. Shobana and Malleshi [25] also observed similar results for crude fibre content in the decorticated finger millet grains. A perusal of proximate composition revealed that chemical constituents except for ADF, NDF and TDN were highly dependent on the seed type (i.e., dehulled seeds and husk) and to a much lesser extent on species (*E. frumentacea* and *E. esculenta*) and cultivars of barnyard millet. The proximate analysis results of dehulled grains and husk indicated their nutritional value and justified their use as food and feed. However, the husk's high ADF and NDF value compared to dehulled grains revealed its poor digestibility and intake by the animals. An adequate amount of physically effective fibre in high-producing dairy cattle is essential for maintaining normal rumen functions, decreasing the risk of metabolic disorders and avoiding suppression of fibre digestion, feed intake, milk production, and alterations in milk composition [26].

We observed low phosphorous in PRJ-1 compared to VL 207 and VL 172, but overall phosphorous values were lower in our study compared to earlier reports in dehulled grains [27]. The Ca content in dehulled grains was also slightly higher than the previous report of Saleh et al. [28] and lower than Mandelbaum et al. [29]. The iron content in cultivars of both the species, i.e., PRJ 1 (13.0 mg/100g) of *E. esculenta*, and VL 207 (13.4 mg/100g) and VL 172 (17.4 mg/100g) of *E. frumentacea* was high. The iron content results agree with the previously observed 15–18 mg/100g range in different studies [29,30]. The manganese and zinc content were higher in *E. esculenta* cv. PRJ-1 than *E. frumentacea* cv. VL 207 and VL 172. The manganese concentration in both species was higher in our study; however, the zinc concentration was low compared to the previous reports [27]. The copper content was very low and agrees with previous reports [27]. The macro-nutrients N and P were present in sufficient quantity in the husk, while micro-nutrients (Ca, Fe, Mn, Zn, Cu) were in traces in the husk compared to dehulled grains in all three cultivars. There is no previous report on mineral estimation of the husk of millets; however, high nitrogen and phosphorous have been reported in rice husk [31]. Low or trace amounts of minerals in husk could be attributed to its function, i.e., protective covering for the whole grain.

To compare the antioxidant activity of Indian and Japanese barnyard millet cultivars, TPC, DPPH and ABTS radical scavenging assays and total antioxidant activity were studied in dehulled grains and husk. All four parameters measured to estimate the antioxidant potential of barnyard millet grains showed that dehulled grains have lower antioxidant metabolites (TPC) and activities than the husk. Dehulling-mediated significant reduction in antioxidant properties of barnyard millet may be attributed to the fact that the often-pigmented seed coat of minor millets is the reservoir of antioxidant metabolites like polyphenols and tannins [25]. Hence, the dehulled grains observed reduced TPC, DPPH and ABTS radical scavenging compared to husk in the present study. These findings are in agreement with the results reported for the dehulled grains of finger millet [25], buckwheat [24], lentil [32] and horse gram [33]. Reduction in TPC may be advantageous because many of the phenolic compounds are considered anti-nutritional factors, and their significant reduction on dehulling may enhance the bioavailability of many essential minerals [34]. The results revealed that antioxidant properties were primarily dependent on grain composition (i.e., dehulled grains and husk), and the differences between species (*E. esculenta* and *E. frumentacea*) and cultivars were marginal and insignificant (Fig. 2).

## 5 Conclusion

The tested cultivars of two cultivated species (*E. frumentacea* and *E. esculenta*) differed significantly in dehulled grain recovery. We conclude that dehulled grain recovery in Japanese barnyard millet, PRJ-1, is significantly less than the Indian barnyard millet cultivars, VL 172 and VL 207. The present investigation describes the physical, chemical and antioxidant properties of dehulled grains and husk of two cultivated species of barnyard millet enlarging the knowledge and providing valuable data for their industrial processing. The comparison of both the species for grain proximate composition, micronutrient profile and antioxidant properties did not reveal significant differences for most of the parameters. Dehulled grains had high protein, ash and essential minerals concentrations, while carbohydrates, fibre and total polyphenols were high in the husk. Breeders can use the data from this study to select desirable genotypes and create novel functional food products using dehulled grains and husk.

## Data availability

Data included in article/supp. material/referenced in article.

## CRediT authorship contribution statement

**Salej Sood:** Conceptualization, Data curation, Formal analysis, Investigation, Methodology, Project administration, Resources, Software, Supervision, Writing – original draft, Writing – review & editing. **Tilak Mondal:** Investigation. **Ramesh S. Pal:** Investigation, Methodology. **Dinesh C. Joshi:** Investigation, Writing – review & editing. **Lakshmi Kant:** Writing – review & editing. **Arunava Pattanayak:** Writing – review & editing.

## Declaration of competing interest

The authors declare that they have no known competing financial interests or personal relationships that could have appeared to influence the work reported in this paper.
